# Hospital process orientation from an operations management perspective: development of a measurement tool and practical testing in three ophthalmic practices

**DOI:** 10.1186/1472-6963-13-475

**Published:** 2013-11-13

**Authors:** Pedro D Gonçalves, Marie Louise Hagenbeek, Jan M H Vissers

**Affiliations:** 1 Institute of Health Policy and Management, Erasmus University Rotterdam, Rotterdam, The Netherlands

**Keywords:** Health services, Process orientation, Process management, Hospital, Measurement tool, Operations management

## Abstract

**Background:**

Although research interest in hospital process orientation (HPO) is growing, the development of a measurement tool to assess process orientation (PO) has not been very successful yet. To view a hospital as a series of processes organized around patients with a similar demand seems to be an attractive proposition, but it is hard to operationalize this idea in a measurement tool that can actually measure the level of PO. This research contributes to HPO from an operations management (OM) perspective by addressing the alignment, integration and coordination of activities within patient care processes. The objective of this study was to develop and practically test a new measurement tool for assessing the degree of PO within hospitals using existing tools.

**Methods:**

Through a literature search we identified a number of constructs to measure PO in hospital settings. These constructs were further operationalized, using an OM perspective. Based on five dimensions of an existing questionnaire a new HPO-measurement tool was developed to measure the degree of PO within hospitals on the basis of respondents’ perception. The HPO-measurement tool was pre-tested in a non-participating hospital and discussed with experts in a focus group. The multicentre exploratory case study was conducted in the ophthalmic practices of three different types of Dutch hospitals. In total 26 employees from three disciplines participated. After filling in the questionnaire an interview was held with each participant to check the validity and the reliability of the measurement tool.

**Results:**

The application of the HPO-measurement tool, analysis of the scores and interviews with the participants resulted in the possibility to identify differences of PO performance and the areas of improvement – from a PO point of view – within each hospital. The result of refinement of the items of the measurement tool after practical testing is a set of 41 items to assess the degree of PO from an OM perspective within hospitals.

**Conclusions:**

The development and practically testing of a new HPO-measurement tool improves the understanding and application of PO in hospitals and the reliability of the measurement tool. The study shows that PO is a complex concept and appears still hard to objectify.

## Background

Hospitals face increasing pressure to reduce costs, improve operations and provide evidence of the quality and efficiency of their organizations [1,2]. This development has led to an increasingly competitive healthcare industry [3]. As competition intensifies, service quality, patient satisfaction and efficient resource management are turning into important indicators for healthcare delivery performance [4,5]. As a result, healthcare organizations are aligning and integrating their care processes horizontally and have started to adopt many of the management principles and techniques that originate from the manufacturing and service industries to respond to and meet the modern healthcare market demands [6-8]. Organizations have come to the conclusion that efficiency as well as quality and service are to be available in processes and not in functional and hierarchical structures [9,10].

As organizations accumulate efforts in process management and process improvements, they gain experience and become more process-oriented. This implicates that the process-oriented approach in some organizations will be more mature than others. But how can a hospital identify whether it is process-oriented or not? And how can a hospital measure the degree of hospital process orientation (HPO)?

### Process orientation and operations management

The objective of this study is to develop and practically test a new measurement tool to assess the degree of process orientation (PO) in hospitals from an operations management (OM) perspective. The OM perspective adds a conclusive scope to existing measurement tools by linking operational processes, the patient care processes, to operations resources, and healthcare delivery patterns [11].

Different authors [11-14] argued that superior operations strategy and effectiveness are related to a sustainable competitive advantage. When superior operations effectiveness is based on skills and capabilities that are embedded in the people and operating processes of the organization and enable them to excel, it not only serves to strengthen a company's existing competitive position, but is also inherently difficult to copy. Operations strategy contributes to the constant reconciliation of market requirements, (i.e. patient demand, evolving regulations, and emerging technologies), and operations resources, (i.e. personnel, equipment, beds, and specialist-time) by shaping the long-term capabilities of the business activities. By designing and delivering healthcare services to meet the needs of patients and other market requirements in the most effective and efficient manner, OM can contribute to the evolving climate in which healthcare services are delivered [11,15].

According to Vissers [16] the identification of key operations corresponds to the first stage in ‘the analysis, design, planning, and control of all the steps necessary to provide a service for a client’. When the elements of operations along with their corresponding duration and workload are added together, it generates the overall set of transforming processes required to deliver a product or service for a client. Consequently, a particular health service can be produced by simultaneously linking the individual diagnostic and therapeutic activities and the resources that they use [11,17]. Vissers & Beech [11] named this a care chain or overall process and defined it as ‘the chain of operations that need to be performed to produce a particular health service.’ The awareness of ‘links’ in the chain of operations enables reflection on key characteristics of patient care processes (e.g. elective, semi-urgent, urgent, complexity, variability, length of process, volume, decoupling points, shared resources, and predictability) and helps to establish appropriate control systems which facilitate decisions about the allocation of resources in order to achieve operational effectiveness. But operational effectiveness cannot be achieved without the support, aid, and drive from corporate and business strategy, i.e. the top and middle management [14].

This is where PO plays a major role. PO has the capacity to function as the foundation for the achievement of operational effectiveness. This foundation is based on the translation and implementation of organizational strategy into operations (processes) by designing the organization around the core processes and rethinking and redesigning the planning and utilization of organizational resources [18]. Therefore, PO establishes an approach to link organizational strategy to implementation within operational processes and it is a manner of putting external emphasis on outcome and customer satisfaction rather than internally driven hierarchical structures or functions [19].

The horizontal, process-oriented, organization emphasizes the need to reshape the internal boundaries and break down vertical silos of the organization in order to make subunits work together horizontally. McCormack & Johnson [20] define PO as *‘An organization that, in all its thinking, emphasizes process as opposed to hierarchies with special emphasis on outcomes and customer satisfaction.’* The design and arrangement of the organization along horizontal workflow processes aiming at linking organizational capabilities to customers and suppliers will improve internal coordination and communication, speed (cycle time), quality, internal and external transparency, financial performance, and increase customer satisfaction [21,22]. By focusing on activities that create value for customers, and view the organization as linked chains of activities, PO delivers a promising solution for a variety of perceived organizational problems in the healthcare industry and other functionally structured organizations [8,23].

Hence, this research offers an approach to address a gap in current research by adding an OM perspective to the organization theoretical approach up to now. This allows the development of a new measurement tool to assess HPO in more operational terms.

### Process orientation in hospitals

Hospitals and healthcare organizations in general have started to move from relatively functional and hierarchical structures to structures focussing on cross-functional teams and flattened organizational structures [24]. The process to become more process-oriented involves all levels of organizational design: governance, structure design, and delivery system [25-27]. The central idea is that the transition to become more process-oriented will lead to more patient-centred care, cost reductions, and quality improvements [22,28]. For years, the organization principles and structural design of hospitals have been labelled as a professional bureaucracy. As a result, the health service delivery processes in hospitals are frequently complex and fragmented across departments because they are being organized according to medical skills or specializations, and not according to the process patients are cared for [29]. This leads to a lack of control and coordination of the care activities within a patient care trajectory, which in turn affects the efficiency and the quality of care delivery [30,31]. The traditional functional structure of hospitals will have to be replaced by a structure which takes a holistic and systematic view of healthcare delivery as a service business process and enables the optimization of healthcare delivery performance [32]. This implies restructuring the health delivery processes in hospitals into integrated care trajectories for nominated patient groups, which are manageable, measurable, and therefore accountable [33,34]. In order to achieve this, the way clinical work is conceived, performed, and organized will have to be altered.

### Existing tools for measuring process orientation

Different authors have addressed the question of conceptualizing and measuring PO. In Table 1 different approaches of measuring PO are summarized. Some authors have addressed it from a management and organization theory, others from an information systems perspective. Kohlbacher & Gruenwald [35] were the first to assess PO from a general, multidimensional perspective and have created and validated a model for the manufacturing industry to assess PO on the basis of seven PO constructs.

**Table 1:** Existing approaches for measuring process orientation

**Author**	**Objective**	**Perspective**	**Distinguished variables or dimensions**	**Applied to hospital setting**
McCormack [41]	Assess the degree at which an organization pays attention to its relevant (core) processes.	Management & Organization theory	Process View; Process Structure; Process Jobs;	No
			Process Measurement & Management;	
			Customer-focused Process Values & Beliefs.	
Andersson et al. [36]	Interpret work and information management in process-oriented healthcare organizations.	Information systems theory	Care activity;	Yes
			Co-ordination activity;	
			Supply activity.	
Reijers [48]	Determine an organizations’ PO prior to BPM system implementation.	Information systems theory	Organizational structure; Focus measurement;	No
			Ownership management; Customer requirements.	
Hammer [49]	Plan and execute process based transformation.	Management & Organization theory	Process enablers (5 elements);	No
			Enterprise capabilities (4 elements).	
Vera & Kuntz [28]	Assess the application of six organizational instruments that lead to a pronounced PO in hospitals.	Management & Organization theory	Process management (4 instruments);	Yes
			Decentralization (2 instruments).	
Gemmel et al. [40] (based on McCormack [41])	Assess the degree at which a hospital pays attention to its relevant (core) processes.	Management & Organization theory	Process View; Process Jobs;	Yes
			Process Measurement & Management.	
Kohlbacher & Gruenwald [35]	Measure the key dimensions of PO on the basis of PO constructs.	Multidimensional	Design & documentation of processes;	No
			Management commitment towards PO;	
			Process owner role;	
			Process performance measurement;	
			Corporate culture in line with process approach;	
			Continuous process improvement methodologies;	
			Process-oriented organizational structure.	

From a management and organization theory perspective, PO is a firm-level construct that supports the streamlining of core processes by more closely linking functional units. From an information systems perspective, PO is based on the information flows associated with the process activities considering that any process with distinct tasks and activities requires information to progress and move forward [36,37]. The information flow in hospitals is dependent on their information system to collect and distribute large amounts of data among various disciplines within a hospital. While interdepartmental dynamics, management and organization theory, and information flows, information systems theory, are important factors for cross-functional process management, these levels of assessing PO do not address the design, planning, and control (improvement) of aligned, integrated and coordinated process activities (OM perspective). This OM perspective is essential for HPO since the design, planning and control of the sequential care activities performed by several medical specialties requires coordination to manage the interdependencies of the process activities [38,39].

Three of the seven measurement tools were applied in hospitals. Andersson et al. [36] referred to the healthcare organizations that have adopted PO as “process-oriented organizational configurations”, Vera & Kuntz [28] stated that hospitals should implement a “process-based organization” to improve their efficiency and Gemmel et al. [40] were the first to use the term ‘hospital process orientation’. The aim of the study of Andersson et al. [36] was to better understand the relationship between work activities and information management in process-oriented healthcare. Therefore the model developed by Andersson et al. [36] is not suitable to measure the alignment, integration, and coordination between process activities because their model is intended to support the development of health information systems (HIS) embedded in process-oriented healthcare work. Vera & Kuntz [28], who were the first to investigate whether the implementation of process-based organizations in hospitals is advisable, used unidimensional measures, e.g. the use of care pathways as an indicator. These organizational items are very broad and do not capture the richness of such a complex concept as PO [35]. As a result, using their indicators, e.g. clinical pathways, and performance-based payment leaves much room for measurement error and does not measure the degree of PO justifiably. Gemmel et al. [40] developed the first measurement tool for HPO and came across other shortcomings by not defining the PO scope. They developed and provided extensive statistical evidence for the validation of a measurement tool for HPO. However, their research and tool did not provide more clarity to the assessment of PO in hospitals. They used a limited PO scope by not including all five dimensions of PO specified by McCormack [41]. The measurement tool was based on the three dimensions - Process View, Process Jobs, and Process Measurement and Management - that were validated by McCormack & Johnson [20] for the e-businesses. But this particular fact is not self-evident in the hospital setting where care processes are delivered by different specialties and where the process management team - Process Structure - and adequate team effort, interdisciplinary communication, and interpersonal skills - Customer-focused Process Values and Beliefs - are crucial factors. Another limitation is that the measurement tool of Gemmel et al. [40] focused on healthcare professionals only, while process management is intended to link operational processes (patient care processes) to direction setting, managerial, and support processes [42].

The present study aims to investigate PO in hospitals from an OM perspective in order to develop a new measurement tool for Hospital Process Orientation. The review of the literature assisted in the identification of gaps within the current research which in turn was used as input for the development of the new HPO measurement tool.

## Methods

### Development of the measurement tool

The conventional research approach is to develop a measurement tool first and subsequently provide statistical evidence for the validation. But given the exploratory nature of this study where the conceptualization and measurement of a rich concept will be executed from a new angle in highly complex institutions with complex environments, it was more appropriate at this stage to practically test the measurement tool than to statistically validate a possible deficient measurement tool.

During the development phase, a new measurement tool based on existing theories, concepts, and measures was developed. McCormack [41] presented PO as a concept which consists of five dimensions: Process View (PV), Process Structure (PS), Process Jobs (PJ), Process Measurement and Management (PMM), and Customer-focused Process Values & Beliefs (PVB). The five dimensions have been used, tested and validated throughout the years [20,43,44]. Through the literature search we were able to specify the theoretical implications of PO for hospital operations and identify a number of constructs to measure PO in a hospital setting. These constructs were further operationalized, using an OM perspective. Based on the definitions of McCormack [41], we conceptualized the five dimensions for the HPO-measurement tool to fit the hospital setting from an OM perspective. Each dimension was operationalized and measured with several items. The foundation of each item is presented in Table 2. Some items find their origin in existing tools, others are based on the constructs identified in our literature search.

**Table 2:** Items’ origin

	**Previous BPO/HPO studies: McCormack**[20]**(M); 10 items Gemmel et al.**[40]**(G); 10 items**	**Operations Management in healthcare: Măruster et al. **[17]**Vissers & Beech **[11]**Langabeer **[15]	**Quality Management in healthcare: Berg et al. **[33]**Van den Heuvel **[46]	**Process Management: Hinterhuber **[45]**Edwards et al. **[47]**Reijers **[48]**Hammer **[49]
**PV**	1 (M + G)	3, 4, 8, 9, 10	6, 7, 11	
	2 (M + G)			
	5 (M + G)			
**PS**	4 (G)	2, 3, 5, 6, 7, 8		1
**PJ**	2 (M + G)			1, 4, 5, 6
	3 (M + G)			
**PMM**	1 (M + G)	3, 5, 6		7
	2 (M + G)			
	4 (M)			
	8 (M + G)			
	9 (G)			
**PVB**	4 (M)			1, 2, 3, 5, 6, 7

#### Process view

Process View is the first step towards PO. Process View involves looking at the organization in a new way by linking business strategy and customer needs to all aspects of process design and management. This requires a clear view on the interrelationships inside and outside the organization and a common language for change management [50]. The PV dimension is to encourage the personnel to view individual actions as links in a chain of events crossing traditional functional barriers – viewing the organization as an integrated set of processes [51].

To accomplish PV, organizations must develop a system architecture in which understanding of the organization and improvement opportunities are established by identifying and mapping the (high-level) business processes [23,52]. Linking business strategy and patient needs to process design, means designing processes to deliver health services to target patient groups.

From an OM perspective, we conceptualized this dimension for the HPO measurement tool as ‘the progress towards organizational focus on integrated business processes by designing, documenting, and managing begin-to-end patient care processes to deliver care to defined target patient groups’. The PV dimension was operationalized and measured with eleven items.

#### Process structure

Following the principle ‘structure follows process’ a process-oriented organization must adapt its Process Structure to the process view [28,35]. The key in process-oriented organizations is to identify how different work activities are holistically accomplished in the organization, to map and manage these cross-functional processes and to use multidisciplinary process teams to carry them out [21]. Working in teams empowers staff, decentralizes decision-making and allows greater learning across the organization. The way a process-oriented organization is structured needs to be supported and promoted by top and middle management also (management commitment to PO). Otherwise the process-oriented initiatives are less likely to secure benefits [47].

Some organizations are not able to align all activities along processes. For that reason, Vanhaverbeke & Torremans [53] suggested a multidimensional structure, combination of functional and process-oriented structure, with process ownership as a solution for organizations, such as hospitals, that cannot adopt a purely process-oriented structure. The existence of process owners is the most visible difference between a process-oriented and a traditional organization [18]. A process owner must have leadership experience and the authority to act in the interest of the process and take all measures necessary to coordinate and improve the business process [45].

The PS dimension was conceptualized from an OM perspective as ‘an organizational structure to coordinate, manage, and improve patient care processes’ and was operationalized and measured with seven items.

#### Process jobs

According to Hammer [49] employees must be skilled in team work, problem solving, process improvement, and decision techniques. Process performers must have appropriate knowledge of how to execute the process in order to be able to implement the process design accordingly [49]. Employees must also embrace the collaboration and the continuous improvement mentality, and feel responsible for these activities.

The knowledge management processes which a process-oriented organization must focus on are those for the creation, transfer and sharing, and the embedding and use of knowledge [19]. Emphasis must be put on cross-skill training and the importance of gaining wider experience by working with different people within different processes. These are both vital factors to align employees’ expectations and aspirations with the process-oriented organization [51]. Furthermore, the organization must have a cadre of experts in change management, process (re-)design, and process improvement methodologies [49].

From an OM perspective, this dimension was conceptualized as ‘the alignment and management of skills, information, knowledge, expertise, traits, and motives of employees to execute and improve patient care processes’. The PJ dimension was operationalized and measured with six items.

#### Process measurement and management

Since a business process can only be controlled and managed if it can be measured, organizations need to implement indicators for performance and take preventive and corrective actions when necessary [45]. Congruence and common focus across separate organizational units can be achieved by focusing measurement on processes instead of functions [54]. Presenting the process performance results to employees allows them to timely react on bad performance of processes or it can motivate employees and improve adherence [35,54].

The PMM dimension was conceptualized from an OM perspective as ‘indicators to periodically measure performance of begin-to-end patient care processes and measures to manage these processes (e.g. review process objectives, and rewards.)’. We operationalized and measured this dimension with nine items.

#### Customer-focused process values and beliefs

Processes are the central core from which business is conducted, as long as they are supported by the people within the organization [55]. Therefore, the cultural fit in process-oriented organizations is an important source of failure or success in PO initiatives [42]. According to Hammer [49] only a culture based on teamwork, willingness to change, customer orientation, personal accountability, and a cooperative leadership style fits organizations applying a process approach.

The PVB dimension was conceptualized from an OM perspective as ‘an organizational culture in line with a process approach’. The PVB dimension was operationalized and measured with six items.

The conceptually developed measurement tool was pre-tested in a non-participating general hospital by an internal management consultant, an ophthalmology department manager and an ophthalmologist. Since we had indications that the importance of each dimension and also each item of PO varies [20,48], we held an OM expert meeting (focus group) to discuss each item and to develop a scoring system for our HPO measurement tool. The items were thoroughly discussed and adapted to the comments. Since our purpose was first to assess the difference in perception between participants, i.e. the degree of PO in hospitals by measuring the scores on different dimensions and not to develop a HPO maturity model at this stage, all experts agreed that each dimension and each item should be considered as equally important.

### Study design and data collection

According to Hellström et al. [8] processes can be studied from an organization, division or department perspective. This research will study processes from the ophthalmology department perspective of three hospitals and try to identify how these processes fit in the organization (hospital) perspective.

We empirically tested the measurement tool by means of a small number of multiple (in-depth) case studies. Case selection should be based on replication logic rather than sampling logic, when building or testing a theory from case studies [56]. For this reason, the criteria for case selection were: 1) predicts similar results and 2) produces contrary results but for predictable reasons. To correspond to this criterion, this research studied the same branch of medicine, i.e. ophthalmology, in a variety of contexts. Ophthalmology is an ideal healthcare practice for the development and testing of the HPO measurement tool due to the fact that it is a comprehensive area of medicine with well-defined processes [57]. The case studies were performed in the ophthalmic practices of three Dutch hospitals: one large university hospital, one large eye specialty hospital and one large general hospital. The choice to include three different types of hospitals into our research is for the purpose of comparing how three different types of hospitals perform on the PO assessment. In addition, applying the HPO measurement tool to different hospital environments will improve its generalizability [58].

Multi-site case studies aiming to identify, describe and link critical variables should be performed by applying structured interviews or survey questionnaires as techniques to collect data [59,60]. Due to the fact that PO is a difficult and complex concept to measure, we chose not to apply the large sample survey method (e-mail or online surveys). The disadvantages and limitations of applying this method did not fit the objective of our study. Since the objective of this study was to identify design requirements for a new measurement tool, to develop it and to give recommendations for further development of the tool, we asked a limited number of participants to empirically test the new HPO measurement tool. For this reason, we only included hospital staff able to oversee and assess the organizational and operational system. The participants in this study are from three disciplines: management, team leaders, and healthcare professionals.

Each of the participating hospitals assigned a contact person to our study. The contact person provided us with a list of potential participants. All of the participants approved their participation after being informed about the purpose of the study, research method, and data processing and management.

We opted for a combined method of collecting data. A combined method is more likely to yield highly productive research output with a lower risk of biased findings [61]. First, the participants filled in the questionnaire which consisted of 39 items. For each item participants were asked to provide the extent to which they agree or disagree with the subject using a four-point Likert scale. The purpose of the empirical study was to test and evaluate the items of the measurement tool and give recommendations for further development of the tool, therefore we did not want the participants to spend too much time in scaling the items and prevent a deliberation of the score (e.g. do I need to score 5 or 6 on a seven point scale).

Afterwards, the filled-in document was discussed with every participant in a semi-structured interview. The combined approach allowed comprehensibility testing of the items (statements) of the HPO measurement tool and the verification of the respondents “perceptual” response. In order to increase contextual understanding and to be able to reflect on and give recommendations for further research and development of the measurement tool, we collected data on thirteen indicators for hospital production, service delivery and availability of resources (Additional file 1).

## Results

### The three case studies

Hospital 1 is a large university hospital, which strives to attain balance between their three organizational objectives: patient care, education, and research. The department of ophthalmology consists of nine ophthalmologists and a total of twelve residents and senior house officers (not specializing). Table 3 shows the number of staff and the supervisee to physician ratio, which is higher than 1 (=1.33) in this hospital. This reflects the mission of medical education within this hospital.

**Table 3:** Staff per hospital (data 2010)

	**Number of Ophthalmologists (FTE)**	**Number of residents and senior house officers not specializing (FTE)**	**Supervisee to physician ratio**
**Hospital 1** University Hospital	9 (8.31)	12 residents and senior house officers (9.78)	1.33
**Hospital 2** Eye Specialty Hospital	30 (24.75)	21 residents and senior house officers (21.9)	0.70
**Hospital 3** General Hospital	8 (*)	4 senior house officers on rotating night shifts (*)	No residency program

(*) missing data.

Hospital 2 is a large hospital specialized in eye care. It is a major referral centre in the Netherlands and it has a workforce of 30 ophthalmologists and a total of 21 residents and senior house officers (not specializing). The supervisee to physician ratio in this hospital is lower than 1 (=0.7). The eye specialty hospital has its own ophthalmic research institute and it has always strived to promote international cooperation between eye hospitals by founding the European Association of Eye Hospitals (EAEH) and the World Association of Eye Hospitals (WAEH).

Hospital 3 is a large general hospital. Eight ophthalmologists are working at this hospital. The night shifts are staffed by four senior house officers (not specializing), who work on rotating night shifts. The residency program in ophthalmology has not been accredited yet, therefore the department of ophthalmology at this general hospital has not been able to train any residents until now.

In 2010, the university hospital performed 24,212 outpatient visits, 2912 surgical procedures/medical interventions with an average of 20 hours a week, and 549 hospital admissions. The percentage of (surgical) procedures that were performed during inpatient admissions was high (=19%). This due to a variety of reasons, but a high case mix index (CMI) was given as the most plausible cause. The university hospital is well experienced in working with and according to processes. In 2008 a project was started to stimulate the design and definition of entire care processes in the form of clinical pathways. The two process descriptions that have been developed for the ophthalmology department are well documented. These (production) characteristics are presented in Table 4.

**Table 4:** (production) Characteristics per hospital (data 2010)

	**Outpatient visits**	**(Surgical) procedures (average hours a week)**	**Hospital admissions (% procedures during admission)**	**Well-defined care processes**
**Hospital 1** University Hospital	24,212	2,912 (20)	549 (18.9%)	2
**Hospital 2** Eye Specialty Hospital	143,636	18,550 (192)	964 (5.2%)	8
**Hospital 3** General Hospital (*)	60,000	2,400 (40)	44 (**)	1

(*) data from 2011 (**) missing data.

The hospital specialized in eye care performed in 2010 a total of 143,636 visits, 18,550 surgical procedures with an average of 192 hours a week, and 964 hospital admissions (only 5% of all procedures were performed during an inpatient admission). This hospital has the most experience with clinical pathways, for more than ten years. The team leaders have to lead care teams, which have been assigned to the various patient groups treated in this hospital (e.g. cataract, strabismus, and macular degeneration etc.). The number of well-defined care processes in this hospital is eight.

Ophthalmology in the large general hospital performed in 2010 a total of 60,000 outpatient visits, 2,400 surgical procedures and 44 inpatient admissions. The Board of Directors of the general hospital is stimulating the development towards a process-oriented organization of care. The ophthalmology department has at the moment only one care process elaborated as care pathway and is planning to elaborate a second care pathway.

In total 26 employees from three disciplines – management, team leaders, and healthcare professionals - participated. The function of each participant within each of the three categories included in this study is presented in Table 5.

**Table 5:** Participants per hospital and per discipline

	**Management**	**Team leaders**	**Healthcare professionals**
	**(n = 8)**	**(n = 8)**	**(n = 10)**
**Hospital 1** University Hospital (n = 9)	- The head of the department,	- 1 unit leader	- 4 ophthalmologists
	- 1 ophthalmology medical-manager	- 1 team leader	
	- 1 staff functionary (quality)		
**Hospital 2** Eye Specialty Hospital (n = 9)	- 1 member of the Board of Directors	- 3 team leaders	- 1 ophthalmologist
	- The head of multidisciplinary treatment teams		- 1 staff nurse
	- 1 staff functionary (OM)		- 1 optometrist/ophthalmic optician
**Hospital 3** General Hospital (n = 8)	- 1 member of the Board of Directors	- 3 unit leaders	- 3 ophthalmologists
	- 1 sector manager		

### Results per hospital

Different types of output resulted from the application of the HPO measurement tool. The first result is the possibility to identify the differences and the areas of improvement within each hospital. Another result is the possibility to understand these differences through the broad notion of the three ophthalmic practices, based on interviewing the participants. Outputs of the application of the HPO measurement tool are provided in Tables 6, 7 and 8.

**Table 6:** Average dimension-scores per hospital with corresponding comprehensibility and reliability percentages

**PO**	**Hospital 1**	**Hospital 2**	**Hospital 3**	**Total**	**Comprehensibility**	**Reliability**
**Dimensions**	**(N = 9)**	**(N = 9)**	**(N = 8)**	**(N = 26)**	**(N = 25)**	**(N = 25)**
**PV**	1.63	1.84	1.83	**1.77**	89%	90%
**PS**	1.73	2.03	1.71	**1.82**	86%	88%
**PJ**	1.87	1.94	2.15	**1.99**	95%	94%
**PMM**	1.48	1.70	1.73	**1.64**	91%	91%
**PVB**	1.76	1.76	2.18	**1.90**	96%	92%
**Total**	**1.69**	**1.85**	**1.92**		**91%**	**91%**

**Table 7:** Internal consistency per dimension

	**Cronbach’s alpha**	**Minimum – Maximum**
	**(N = 26)**	**Corrected item-total correlation**
Process View (11 items)	0.768	0.280 – 0.625
Process Structure (7 items)	0.760	0.199 – 0.622
Process Jobs (6 items)	0.686	0.226 – 0.700
Process Measurement and	0.840	0.321 – 0.719
Management (9 items)		
Customer-focused Process	0.781	0.180 – 0.812
Values and Beliefs (6 items)		

**Table 8:** Average dimension-scores per hospital/per discipline

	**Hospital 1**			**Hospital 2**			**Hospital 3**		
**PO**	**Management**	**Team leaders**	**Healthcare professionals**	**Management**	**Team leaders**	**Healthcare professionals**	**Management**	**Team leaders**	**Healthcare professionals**
**Dimensions**	**(n = 3)**	**(n = 2)**	**(n = 4)**	**(n = 3)**	**(n = 3)**	**(n = 3)**	**(n = 2)**	**(n = 3)**	**(n = 3)**
**PV**	1.94	1.36	1.59	2.24	1.85	[1.42]	2.14	[1.67]	[1.70]
**PS**	1.95	1.57	[1.68]	2.48	1.81	[1.81]	2.14	1.62	1.38
**PJ**	1.94	2.00	1.67	2.33	2.06	1.44	2.33	2.39	1.72
**PMM**	1.74	1.17	[1.53]	2.22	[1.52]	1.37	1.83	1.81	1.56
**PVB**	1.94	1.58	1.75	2.06	1.72	[1.50]	2.58	2.11	1.83

[Scores in brackets indicate that there were large differences in the item scores between participants within the discipline].

The nominal Likert scale was converted into values of 0–3, retaining, respectively, the ranking of responses with 0 for “completely disagree” and 3 for “completely agree”.

The total average dimension scores of all three hospitals were below 2, between ‘agree’ and ‘disagree’. Hence, from the participants’ perspective all three hospitals performed moderately in terms of process orientation. The PMM scored the lowest, 1.64 on a scale from 0 to 3. The PJ and PVB dimensions scored the highest, respectively 1.99 and 1.90. The PV and PS dimensions scored 1.77 and 1.82, which is relatively low.

The low PMM score was probably due to a lack of outcome indicators to measure 1) the performance of begin-to-end care processes (entire patient trajectories) or 2) the use of mainly financial or production measures for organizational performance. When asked to name a few indicators a number of participants gave examples of external indicators, for instance from the Healthcare Transparency Programme (Zichtbare Zorg) or the Dutch Healthcare Inspectorate. These external indicators are in the first place meant to measure the quality of data registry and in second place to measure the performance of processes (process indicators). Hence, these indicators do not measure the performance of begin-to-end care processes and have little input for the improvement of internal care processes as both measures are not able to predict the results of the care processes. Financial and production performance come from the results of management actions and organizational performance. They are not the results of the core processes in healthcare, the clinical interventions. Furthermore, organizational performance is not only dependent of financial and production objectives, but also of outcomes on how well the organization adapts to their external environment or the service level it offers to its clients.

The low score on the PV en PS dimensions is probably due to a limited amount of well-defined care processes within Hospital 1 and 3, as presented in Table 4. According to Cinquini et al. [52] the process view will have to be based on a highly detailed process model. The process model has to indicate the system of rules and responsibilities of actors across the organization, facilitate the assessment of resource consumption accurately, facilitate allocation of resources among units by exemplifying a profile capacity usage, and enable the realization of a detailed variance analysis [52]. The process models or clinical pathways that are being developed in hospital 1 and 3 were not able to provide this kind of support to hospital processes. Hospital 2 has a longer experience in process mapping and improvement, and has a team based organizational structure. As expected, hospital 2 scored the highest on the PS dimension and also very high on the PV and PMM dimension.

Since hospitals are a working environment for well trained and licensed medical professionals and a multidisciplinary approach, teamwork, and a patient-focused culture have been applied and exercised for years in hospitals in the Netherlands, we expected high scores on the PJ and PVB dimensions. A result that was not foreseen was that Hospital 3 came out with the highest average score. There was no evidence found in other sources of information to back-up this result.

Table 6 also reports on the comprehensibility and reliability degree per dimension. The comprehensibility degree was operationalized as the percentage of the number of items stated as clear by the participants, and the reliability degree as the percentage of the number of unchanged responses after the interview. We were not able to interview one of the participants from hospital 1. Therefore, we only included the item scores of the non-interviewed participant (staff functionary from hospital 1) as research data and treated the comprehensibility and reliability scores as missing values. The items in the HPO measurement tool performed excellently on the measures to assess the comprehensibility and reliability, both were above 90%.

In addition, the items in the measurement tool were tested for internal consistency using the Cronbach’s alpha based on the scores of the 26 participants [62]. Table 7 shows that all items scored a satisfactory α value between 0.7 and 0.9 [63], except the Process Jobs dimension. The questionable α value for the PJ dimension is due to the fact that there were two items in the PJ dimension, PJ1 and PJ4, which had a low corrected item-total correlation value, respectively 0.226 and 0.276. This value should be ideally above 0.3 [64]. The reason for the low item-total correlation is that these two statements were not specific enough. Participants commented that teamwork is not always necessary and that there are plenty of employees with expertise in the improvement of care processes in their hospital, but they are always busy and are not always available. These two items were specified by adding “teamwork within care processes” to item PJ1 and “employees available in our hospital” to item PJ4. In the other dimensions there were only single items which had an item-total correlation value of less than 0.3. The items PV1 (0.280), PS4 (0.199), and PVB1 (0.180) were thoroughly revised after the interviews. Actually, based on the outcomes of the interviews and the internal consistency, comprehensibility and reliability tests, all items were rechecked and almost all of the items were revised. Two items were added, after the experiences with the original survey; ‘PV7: There are no misunderstandings in the interpretation of a care description between hospital staff’ and ‘PS6: Each care process is aligned and coordinated between the different healthcare professionals involved in the entire care process’. One item was replaced from the PV dimension to the PVB dimension; ‘PVB4: Hospital staff on all levels communicates in terms of processes, teams, and process performance’. Within every dimension the order of the items was changed into a more logical sequence that matched the expectations of the participants. Also the definitions of all the dimensions were slightly adjusted. Additional file 2 shows the definitions and the complete list of items of the updated HPO measurement tool.

Furthermore, we changed the scale to a 7-point Likert scale. In our study we used a 4-point Likert scale for two reasons: 1) we did not want a neutral scale item in our Likert-type scale, and 2) we did not want the participants to spend too much time in scaling the items. However, for further testing a 4-point Likert scale is too narrow. It will not reveal patterns of the scaled items that would indicate significant differences.

The results in Table 8 also lead to a conclusion that the PO perception seems to be higher for disciplines that are more distant from clinical practice. This conclusion is true for two hospitals except for the university hospital. In general, the average PO score of healthcare professionals was usually lower than the average score of the team leaders. In the university hospital it was the other way around.

A plausible explanation for this is the complexity of patient care in a university hospital. In order to be able to expand medical knowledge and increase performance skills university hospitals have a large number of physicians with in-depth expertise in increasingly narrow fields of clinical practice [65]. This subspecialization and the complex medical science and technology applied in university hospitals makes it difficult for team leaders to concentrate on the development and delivery of best cost-effective care pathways for defined patient groups [66]. Consequently, team leaders in a university hospital may not perceive a higher level of process orientation.

Another explanation could be the role division between management, team leaders, and physicians in university hospitals. In the studied university hospital, management and physicians are closely related and team leaders do not perform a linking role between management and doctors. Therefore, team leaders may have a limited overview of the entire care process compared to healthcare professionals working within the care process.

In most cases the perception of management participants had a larger positive influence than the perception of the other categories, and their overall score played a dominant role in the higher average score on the PO dimensions. This applies especially to Hospital 3.

Scores between brackets in Table 8 indicate that there were large differences in the item scores between participants within the same discipline. The reason for the large differences were that the participants did not have the same opinion on, for instance, what a care process description exactly is, how detailed it should be and whether the care process description needs to be accessible to all employees involved in the care process (PV), the existence of a process owner as indicated in the measurement tool (PS), whether the performance of entire patient trajectories were measured by means of indicators (PMM), and whether a continuous improvement culture existed within their hospital (PVB).

## Discussion

This research improves the understanding and application of PO in hospitals. The developed measurement tool can not only be used to assess the degree of each PO dimension in a hospital, but can also be used to review the internal progress of process-oriented organization of hospital care. The case studies allowed a deep understanding of the nature and complexity of the complete phenomenon (PO), which could be translated into recommendations to improve the measurement tool for measuring hospital process orientation. We will first discuss below the strengths and limitations of our approach, and then formulate the research recommendations.

### Strengths and limitations

In general, there were indications that the measurement tool is vulnerable to over-positive or over-negative responses due to own interests, motivation or expectations of the respondent. Therefore, the measurement tool performs less well in comparing hospitals on PO, as the subjective answers of respondents can be influenced by the positive attitude of the management of the hospital that is not counterbalanced by actual development of process orientation in for instance care pathways. Apart from this, the perception of the respondent on PO has his own impact. Indirectly, the measurement tool is measuring the differences in perception between respondents. The perception on PO is an interesting factor. It says something about the vision of the organization on PO and to what extent PO is disseminated in the organization. On the other hand, to measure the degree of PO in a hospital the measurement tool needs to measure an ‘objective’ degree of PO. The combined approach followed with an interview and discussion of the filled-in questionnaire allowed reflection on the respondents answer and if necessary to correct the first response into a more deliberated answer. As a supplement, quantitative data on thirteen indicators for hospital production, service and capacity was collected to increase contextual understanding and to be able to reflect on and give recommendations for further research and development of the measurement tool.

An important limitation of this study is the small sample size. However, given the purpose of this study, the sample size was satisfactory. The objective was to practically test the measurement tool and its content (items), as a first step towards statistical testing of validity and measurement precision.

### Practical implications and future developments

There are a number of areas to improve the HPO measurement tool for future research. First of all, the use of perceptions alone to assess the internal degree of PO leads to a subjective measurement procedure that can be manipulated. Therefore, the measurement tool will have to be complemented by metrics for each dimension. Research will have to be conducted to search for metrics to identify and measure each dimension objectively. An example of such a metric for the Process View dimension can be: ‘the percentage of the total patient population treated according to a well-defined and agreed upon care process’. Other examples are: ‘the percentage of care process ownership (% of care processes assigned to a process owner)’, ‘teamwork evaluation’, ‘access time, amount of medical errors, and patient experience’, and ‘internal transparency of objective realization progress’. The use of supplementary metrics in combination with the HPO measurement tool will increase the reliability of the output of the internal PO assessment.

Future studies on the statistical validation of the HPO measurement tool should focus on the reliability, construct validation, and convergent & divergent validity of the instrument. This is only possible by examining and assessing the variability and internal consistency of the measures with a much larger sample size. Chen et al. [67] provided an integrative framework for multi-level construct validation, which can be used as a guide through the statistical validation process.

We would like to emphasise that a degree of PO is merely an indication for how the structure design and work within an organization is organized. High scores on each dimension of the measurement tool may be expected to have positive effects on the design, planning, and control of the processes within the organization, and organizations will not necessarily have the best performances, like the shortest throughput time as possible. Thus, a high score on the degree of PO will not guarantee the best hospital performance. Research will have to be conducted to link the PO dimensions or defined PO constructs (e.g. process owner role, and allocation and assignment of resources to care processes) to hospital performance (e.g. patient outcomes (group-level), and throughput time).

Another issue for future research is the complexity of measuring hospital performance, since hospital success and continuity is not only dependent of clinical interventions and financial and production objectives, but also on how well the organization adapts to their external environment or the service level it offers to its clients. For an extended debate on how to measure and manage healthcare performance, the articles of Dey et al. [68], Keung [69], and Bouckaert & Halligan [70] provide support.

To a different degree and for distinctive reasons all three hospitals had some difficulties with the PV and PMM dimension. These two dimensions are at the core of process orientation of an organization. Hellström et al. [8] already presented this problem as: “the hospital as an organization itself in many ways becomes an obstacle to the achievement of a process-oriented management style”. To acquire a process-oriented management style, consensus for each care process has to be reached among a variety of healthcare professionals, managers, staff functionaries, and patients in a particular healthcare setting. Subsequently, the hospital will have to adapt existing medical guidelines to local circumstances and preferences in order to define a care process description, which is evidence-based and adjusted to the local care setting. Achieving consensus in a local care setting is one of the biggest obstacles to acquire an adequate process view in hospitals. Lillrank & Liukko [71] classified processes in healthcare into three groups: standard, routine, and non-routine. Our recommendation would be to start with the standard processes and the routine processes first. Once consensus is reached, organizations will have to take the next step and compose indicators to measure the performance and outcomes of begin-to-end care processes.

Finally, our results already indicated an unusual effect of university hospitals on the PO score. Therefore, the historic debate on methodological issues concerning comparative studies of hospital-organizations [72,73], is also valid for the generalizability of our findings, measurement tool, and future research. The modern roles and functions of hospitals nowadays have made it even more difficult to make use of simple classifications when studying hospital-organizations [74].

Our results show that discipline may have an effect on the PO score. The perception of management was consistently higher in our study. Their score had a large positive influence on the average PO score (the further away from the care process, the higher the PO score).

Another variable is the rather mono-disciplinary nature of ophthalmology as a specialty. Ophthalmology, and other specialties such as dermatology, orthopaedics, and ENT (ear, nose, and throat) have more routine care processes and these specialties do not work intensively with other specialties [71,75,76]. The delimited area of medicine may reduce the complexity of the ophthalmology departments which could have a positive effect on the level of PO within the departments and lead to a higher PO score. An approach to deal with this is to distinguish the specialties with standard and routine processes and less interaction with other specialties from specialties with non-routine processes and more interactions and collaboration with other specialties such as oncology, cardiology, neurology, and gastroenterology. All these variables should be considered when applying this measurement tool in hospital departments and hospitals in general, since they may have an effect on the average PO score.

## Conclusions

The purpose of this study is to investigate PO in hospitals from an OM perspective in order to develop and practically test a measurement tool to measure HPO in operations management terms. The intention to develop such a measurement tool is to measure the design, planning, and control of aligned, integrated, and coordinated cross-functional processes in hospitals.

Many hospitals are in the process of restructuring and introducing new coordination and skill mix mechanisms in order to integrate units (clinical integration), introduce multidisciplinary teams, and resource pooling, which are the basis of process orientation [31,66]. By applying this measurement tool hospitals can classify the perception of process orientation within their organization on the basis of mean values. Consequently, hospitals will be able to identify strong areas of PO within their organizations and areas for improvement.

In this manner, the application of this measurement tool enables hospitals to measure the effects of the change processes they applied to become more process-oriented and evaluate how they are evolving towards process-oriented care delivery.

The results from the practical testing of the measurement tool show that the developed tool was able to measure the differences between hospitals from the proposed OM perspective to a certain degree. The study suggests that by measuring the 41 items, a hospital can assess the degree of PO of the organization from an OM perspective. The total of 41 items reflects the magnitude and complications to become a process-oriented hospital. Nevertheless, hospitals will have to use the whole range of items and include distinct disciplines in order to get a complete assessment. In addition, metrics should be used to objectify the perception of the participants.

### Ethics statement

In this research no human subjects (including human material or human data) were involved. Therefore, neither approval by an appropriate ethics committee nor informed consent from participants to participate in the study was obtained.

## Abbreviations

CMI: Case mix index; EAEH: European association of eye hospitals; HPO: Hospital process orientation; OM: Operations management; PJ: Process jobs; PMM: Process measurement and management; PO: Process orientation; PS: Process structure; PV: Process view; PVB: Customer-focused process values and beliefs; WAEH: World association of eye hospitals.

## Competing interests

The authors declare that they have no competing interests.

## Authors’ contributions

This manuscript is based on the master thesis of PDG. All authors contributed to the conception and design of the study. Literature search was done by PDG. All authors read all relevant articles and contributed equally to the development of the measurement tool. PDG was responsible for the practical testing. All authors were responsible for analyzing the data. JMV supervised the master thesis of PDG and the content of the article. MLH was responsible for drafting the manuscript and preparation for submission to *BMC Health Services Research*. All authors read and approved the final manuscript.

## Pre-publication history

The pre-publication history for this paper can be accessed here:

http://www.biomedcentral.com/1472-6963/13/475/prepub

## Supplementary Material

Additional file 1 Indicators for hospital production, service, and available resources. Click here for file

Additional file 2 The HPO-measurement tool after testing. Click here for file

## References

[B1] Mango PD Shapiro LA Hospitals get serious about operations McKinsey Q 2001 2 74 85

[B2] Kujala J Lillrank P Kronström V Peltokorpi A Time-based management of patient process J Health Organ Manag 2006 20 6 512 524 10.1108/14777260610702262 17168103

[B3] Swayne LE Duncan WJ Ginter PM Strategic management of health care organizations 2008 6 San Francisco, CA: Jossey-Bass

[B4] McDermott C Stock GN Hospital operations and length of stay performance Int J Oper Prod Manag 2007 27 9 1020 1042 10.1108/01443570710775847

[B5] Cowing M Davino-Ramaya CM Ramaya K Szmerekovsky J Health care delivery performance: service, outcomes, and resource stewardship Perm J 2009 13 4 72 77 20740107 10.7812/tpp/08-100 PMC2911834

[B6] Walston SL Lazes P Sullivan PG Improving hospital restructuring: lessons learned Health Care Manage Rev 2004 29 4 309 319 10.1097/00004010-200410000-00007 15600109

[B7] Langabeer JR DelliFraine JL Heineke J Abbass I Implementation of lean and six sigma quality initiatives in hospitals: a goal theoretic perspective Oper Manag Res 2009 2 1-4 13 27 10.1007/s12063-009-0021-7

[B8] Hellström A Lifvergren S Quist J Process management in healthcare: investigating why it’s easier said than done J Manuf Technol Manag 2010 21 4 499 511 10.1108/17410381011046607

[B9] Hammer M Reengineering work: don’t automate, obliterate Harv Bus Rev 1990 68 4 104 112

[B10] Davenport TH Short JE The new industrial engineering: information technology and business process redesign Sloan Manage Rev 1990 31 4 11 28

[B11] Vissers J Beech R Health Operations Management. Patient Flow Logistics in Health Care 2005 London: Routledge

[B12] Hayes RH Pisano GP Beyond world-class: the new manufacturing strategy Harv Bus Rev 1994 77 86

[B13] Hayes RH Upton DM Operations-based strategy Calif Manage Rev 1998 40 4 8 25 10.2307/41165962

[B14] Slack N Lewis M Operations Strategy 2007 5 Essex: Pearson Education Limited

[B15] Langabeer JR Health Care Operations Management. A quantitative approach to business and logistics 2008 Boston: Jones and Bartlett Publishers

[B16] Vissers JMH Patient Flow based Allocation of Hospital Resources PhD thesis 1994 Eindhoven: University of Technology 10.1093/imammb/12.3-4.259 8919562

[B17] Măruster L Weijters A de Vries G van den Bosch A Daelemans W Logistic-based patient grouping for multi-disciplinary treatment Artif Intell Med 2002 26 1 87 107 12234719 10.1016/s0933-3657(02)00054-4

[B18] Hammer M Stanton S How process enterprises really work Harv Bus Rev 1999 77 6 108 118 10662000

[B19] Armistead C Knowledge management and process performance J Knowl Manag 1999 3 2 143 154 10.1108/13673279910275602

[B20] McCormack KP Johnson WC Business process orientation: Gaining the e-business competitive advantage 2001 London: St. Lucie Press

[B21] Anand N Daft RL What is the right organization design? Organ Dyn 2007 36 4 329 344 10.1016/j.orgdyn.2007.06.001

[B22] Kohlbacher M The effects of process orientation: literature review Bus Process Manag J 2010 16 1 135 152 10.1108/14637151011017985

[B23] Hellström A Eriksson H Are you viewing, mapping or managing your processes? TQM J 2008 20 2 166 174 10.1108/17542730810857390

[B24] Vos L van Oostenbrugge RJ Limburg M van Merode GG Groothuis S How to implement process-oriented care Accred Qual Assur 2009 14 5 13 10.1007/s00769-008-0436-0

[B25] Degeling PJ Maxwell S Hunter DJ Making clinical governance work Br Med J 2004 329 7467 679 681 10.1136/bmj.329.7467.679 15374921 PMC517655

[B26] Lega F Organisational design for health integrated delivery systems: Theory and practice Health Policy 2007 81 2 258 279 16846661 10.1016/j.healthpol.2006.06.006

[B27] Vos L Dückers MLA Wagner C van Merode GG Does case-mix based reimbursement stimulate the development of process-oriented care delivery? Health Policy 2010 98 1 74 80 10.1016/j.healthpol.2010.06.005 20605051

[B28] Vera A Kuntz L Process-based organization design and hospital efficiency Health Care Manage Rev 2007 32 1 55 65 10.1097/00004010-200701000-00008 17245203

[B29] Lee JG Clarke RW Restructuring improves hospital competiveness Healthc Financ Manage 1992 46 11 30 37 10145711

[B30] Nyssen AS Coordination in hospitals: organized or emergent process? Cogn Tech Work 2007 9 3 149 154 10.1007/s10111-006-0053-9

[B31] Vos L Chalmers SE Dückers MLA Groenewegen PP Wagner C van Merode GG Towards an organisation-wide process-oriented organisation of care: a literature review Implement Sci 2011 6 8 1 14 21247491 10.1186/1748-5908-6-8 PMC3035025

[B32] Parnaby J Towill DR Enabling innovation in health-care delivery Health Serv Manage Res 2008 21 3 141 154 10.1258/hsmr.2007.007014 18647942

[B33] Berg M Schellekens W Bergen C Bridging the quality chasm: integrating professionals and organizational approaches to quality Int J Qual Health Care 2005 17 1 75 82 10.1093/intqhc/mzi008 15668314

[B34] Jain AK Thompson JM Kelley SM Schwartz RW Fundamentals of service lines and the necessity of physician leaders Surg Innov 2006 13 2 136 144 10.1177/1553350606291044 17012155

[B35] Kohlbacher M Gruenwald S Process orientation: conceptualization and measurement Bus Process Manag J 2011 17 2 267 283 10.1108/14637151111122347

[B36] Andersson A Hallberg N Timpka T A model for interpreting work and information management in process-oriented healthcare organisations Int J Med Inform 2003 72 1 47 56 14644306 10.1016/j.ijmedinf.2003.09.001

[B37] Berente N Vandenbosch B Information flows and business process integration Bus Process Manag J 2009 15 1 119 141 10.1108/14637150910931505

[B38] Malone T Crowston K The interdisciplinary study of coordination ACM Comput Surv 1994 26 1 87 119 10.1145/174666.174668

[B39] Mans RS Workflow Support for the Healthcare Domain PhD thesis 2011 Eindhoven: University of Technology

[B40] Gemmel P Vandaele D Tambeur W Hospital Process Orientation (HPO): the development of a measurement tool Total Qual Manag 2008 19 12 1207 1217 10.1080/14783360802351488

[B41] McCormack KP Johnson WC Business process orientation: Gaining the e-business competitive advantage 2001 London: St. Lucie Press

[B42] Armistead C Machin S Implications of business process management for operations management Int J Oper Prod Manag 1997 17 9 886 898 10.1108/01443579710171217

[B43] Lockamy A III McCormack K The development of a supply chain management process maturity model using the concepts of business process orientation Supply Chain Manag Int J 2004 9 4 272 278 10.1108/13598540410550019

[B44] McCormack K Willems J Bergh J Deschoolmeester D Willaert P Štemberger M Škrinjar R Trkman P Ladeira M Oliveira MPV Vuksic V Vlahovic N A global investigation of key turning points in business process maturity Bus Process Manag J 2009 15 5 792 815 10.1108/14637150910987946

[B45] Hinterhuber HH Business process management: the European approach Bus Change Re-engineering 1995 2 4 63 73

[B46] Van den Heuvel J The effectiveness of ISO 9001 and Six Sigma in Healthcare PhD thesis 2007 Rotterdam: Erasmus University

[B47] Edwards C Braganza A Lambert R Understanding and managing process initiatives: a framework for developing consensus Knowl Process Manag 2000 7 1 29 36 10.1002/(SICI)1099-1441(200001/03)7:1<29::AID-KPM80>3.0.CO;2-0

[B48] Reijers HA Implementing BPM systems: the role of process orientation Bus Process Manag J 2006 12 4 389 409 10.1108/14637150610678041

[B49] Hammer M The process audit Harv Bus Rev 2007 85 4 111 121 17432158

[B50] McCormack K Rauseo N Building an enterprise process view using cognitive mapping Bus Process Manag J 2005 11 1 63 74 10.1108/14637150510578737

[B51] Armistead C Principles of process management Manag Serv Qual 1996 6 6 48 52 10.1108/09604529610149239

[B52] Cinquini L Vitali PM Pitzalis A Campanale C Process view and cost management of a new surgery technique in hospital Bus Process Manag J 2009 15 6 895 919 10.1108/14637150911003775

[B53] Vanhaverbeke W Torremans H Organizational structure in process-based organizations Knowl Process Manag 1999 6 1 41 52 10.1002/(SICI)1099-1441(199903)6:1<41::AID-KPM47>3.0.CO;2-4

[B54] Hammer M The 7 deadly sins of performance measurement and how to avoid them MIT Sloan Manag Rev 2007 48 19 28

[B55] Jeston J Nelis J Management by Process: A Roadmap to Sustainable Business Process Management 2008 Oxford: Elsevier

[B56] Voss C Tsikriktsis N Frohlich M Case research in operations management Int J Oper Prod 2002 22 2 195 219 10.1108/01443570210414329

[B57] de Korne DF Sol KJ van Wijngaarden JD van Vliet EJ Custers T Cubbon M Spileers W Ygge J Ang CL Klazinga NS Evaluation of an international benchmarking initiative in nine eye hospitals Health Care Manage Rev 2010 35 1 23 35 10.1097/HMR.0b013e3181c22bdc 20010010

[B58] Wacker JG A definition of theory: research guidelines for different theory-building research methods in operations management J Oper Manag 1998 16 361 385 10.1016/S0272-6963(98)00019-9

[B59] Handfield RB Melnyk SA The scientific theory-building process: a primer using the case of TQM J Oper Manag 1998 16 4 321 339 10.1016/S0272-6963(98)00017-5

[B60] Stuart I McCutcheon D Handfield R McLachlin R Samson D Effective case research in operations management: a process perspective J Oper Manag 2002 20 419 433 10.1016/S0272-6963(02)00022-0

[B61] Boyer KK Swink ML Empirical elephants—Why multiple methods are essential to quality research in operations and supply chain management J Oper Manag 2008 26 337 348 10.1016/j.jom.2008.03.001

[B62] Cronbach LJ Coefficient alpha and the structure of tests Psychometrika 1951 16 3 297 334 10.1007/BF02310555

[B63] Tavakol M Dennick R Making sense of Cronbach’s alpha Int J Med Educ 2011 2 53 55 10.5116/ijme.4dfb.8dfd PMC4205511 28029643

[B64] Everitt BS The Cambridge Dictionary of Statistics 2002 2 Cambridge: Cambridge University Press

[B65] Griffin DJ Hospitals: What They Are and How They Work 2012 4 Burlington, MA: Jones & Bartlett

[B66] Lega F DePietro C Converging patterns in hospital organization: beyond the professional bureaucracy Health Policy 2005 74 3 261 281 10.1016/j.healthpol.2005.01.010 16226138

[B67] Chen G Mathieu JE Bliese PD A framework for conducting multilevel construct validation Res multi-level Issues 2004 3 273 303

[B68] Dey KD Hariharan S Despic O Managing healthcare performance in analytical framework Benchmarking Int J 2008 15 4 444 468 10.1108/14635770810887249

[B69] Keung P Process performance measurement system: a tool to support process-based organizations Total Qual Manag 2000 11 1 67 85 10.1080/0954412007035

[B70] Bouckaert G Halligan J A framework for comparative analysis of performance management Paper presented at Study Group on Productivity and Quality in the Public Sector 2006 Milan: Conference of European Group of Public Administration, Università Bocconi 6 9

[B71] Lillrank P Liukko M Standard, routine and non-routine processes in healthcare Int J Health Care Qual Assur 2004 17 1 39 46 10.1108/09526860410515927 15046472

[B72] Alford RR Research note: problems of data and measurement in interorganizational studies of hospitals and clinics Adm Sci Q 1974 19 4 485 490 10.2307/2391805

[B73] Graeff CL Some methodological issues concerning comparative hospital-organization studies Acad Manag Rev 1980 5 4 539 548 10248800

[B74] McKee M Healy J Hospitals in a changing Europe 2002 Buckingham: Open University Press

[B75] Schein OD Katz J Bass EB Tielsch JM Lubomski LH Feldman MA Petty BG Steinberg EP The value of routine preoperative medical testing before cataract surgery N Engl J Med 2000 342 3 168 175 10.1056/NEJM200001203420304 10639542

[B76] Larsen T Evidence on the efficacy of integrated care Int J Health Care Deliv Reform Initiat 2009 1 3 71 88

