# Supplementary material for: Hospital process orientation from an operations management perspective: development of a measurement tool and practical testing in three ophthalmic practices
Source: BMC Health Serv Res. 2013 Nov 13;13:475. doi: 10.1186/1472-6963-13-475 (PMC3831252; doi:10.1186/1472-6963-13-475)
Supplement: Additional file 1 — Indicators for hospital production, service, and available resources. [file 1472-6963-13-475-S1.doc]

**Indicators for hospital production, service and available resources.**

| **Production metrics *** |
| --- |
| 1. Number of initial visits (new patients) |
| 1. Number of follow-up visits |
| 1. Number of (surgical) procedures |
| 1. Number of hospital admissions |

| **Service attributes*** |
| --- |
| 1. Waiting time for first outpatient visit (access time) |
| 1. Waiting time for operation/procedure. |

* Production metrics and service attributes were specified for eight patient groups: cataract, glaucoma, macular degeneration, strabismus, cornea, vitreoretinal surgery, diabetic retinopathy and other groups.

| **Available capacity/resources** |
| --- |
| 1. Number of ophthalmologists |
| 1. Number of residents and senior house officers |
| 1. Number of staff formations (hospital ward and outpatient clinic) |
| 1. Number of beds (surgical day care and hospital ward) |
| 1. Maximum number of parallel clinic sessions |
| 1. Average number of outpatient clinic session hours per week |
| 1. Average number of operating theatre hours per week |
