# Supplementary material for: Hospital process orientation from an operations management perspective: development of a measurement tool and practical testing in three ophthalmic practices
Source: BMC Health Serv Res. 2013 Nov 13;13:475. doi: 10.1186/1472-6963-13-475 (PMC3831252; doi:10.1186/1472-6963-13-475)
Supplement: Additional file 2 — The HPO-measurement tool after testing. [file 1472-6963-13-475-S2.doc]

# The HPO-measurement tool after testing

Process View (PV): ‘*personnel on all levels of the organization have knowledge and understanding of process steps, activities and tasks of cross-functional patient care processes. The care process in the hospital includes all contacts with the patient and all activities for the patient between referral to the time of discharge’.*

1. I have insight/understanding in all of the care process steps that a patient goes through in our hospital.
2. Personnel have insights/understanding in the connection/links between departments, which are required for the care process.
3. We differentiate patient groups in our patient inflow.
4. In our hospital, we have a separate care process description for each patient group.

(N. B. a care protocol/medical guideline is not a process description)

1. The steps in our care processes have been described and are fully documented.
2. Our care process descriptions are accessible to all employees involved in the care process.
3. There are no misunderstandings in the interpretation of a care description between the hospital staff.
4. Our care process descriptions contain information on the required resources for each process step.
5. Our care process descriptions also contain information on the current throughput time and waiting times of each process step.
6. Our care processes contain service goals (e.g. provision of information to patients, acceptable waiting times, patient interaction et cetera).
7. Care process descriptions are adjusted if necessary.

Process Structure (PS): *‘a work system which focuses on processes and teams rather than on departments. The Process Structure acts as an organizational framework for the other dimensions’.*

1. The management of our hospital actively stimulates a process-driven organization/mentality.
2. The design and management of care processes is an important entity within our hospital.
3. We apply a systematic approach to improve our processes.
4. To improve our care processes we involve staff from all concerned departments and patients.
5. A multidisciplinary team has been established for each care process.
6. Each care process is aligned and coordinated between the different health care professionals involved in the entire patient care process.
7. For the care process of each patient group there is a designated person/employee who has the responsibility and authority to monitor the care process and maintain it up-to-date. (We called this a process owner)
8. The management of our hospital has been proactive in responding to impacts of external developments (i.e. demographic, technological, cooperation) for the internal practice of our care processes.

Process Jobs (PJ): *‘jobs focus on entire care processes and have horizontal responsibilities. Collaboration to achieve common goals to improve the patient care process is important’*.

1. In our hospital, teamwork within care processes is an element of evaluation during performance evaluations.
2. Employees feel responsible for the organization of care processes.
3. Employees pay attention to process improvement in their daily work.
4. There are plenty of employees available in our hospital with expertise in the improvement of care processes.
5. The process owner assesses and ensures that a care process fulfills the needs and wishes of patients.
6. The team of employees is able to guarantee the continuity of the care process during absence (vacation, sick leave etc.) of colleagues.

Process Measurement & Management (PMM): *Indicators to continuously evaluate the performance of all care processes, the application of process improvement techniques to manage processes and reward/providing incentives for improvements on process-oriented organization of care’.*

1. The performance of each care process is measured by indicators regularly.

(N. B. by performance we mean patient satisfaction, clinical outcomes, waiting and throughput times et cetera. Not just production/financial performance)

1. Performance measurements taken cover the entire care process (both ambulatory and clinical phase).
2. Care processes are evaluated periodically to assess whether they still fulfill the needs and desires of our patients.
3. The available resources are distributed across the multiple patient groups to ensure a smooth performance of care processes.
4. Information on the use of resources during the last period is considered when reallocating resources per patient group.
5. Process objectives are altered and reformulated when strategic goals are revised.
6. In our hospital, the performance of teams/teamwork is measured when effectuating the care processes.
7. In our hospital, we measure whether goals related to care process quality and efficiency are achieved.
8. Processes are modified if the objectives in terms of care process quality and efficiency are not being achieved.

Customer-focused Process Values & Beliefs (PVB): *‘Commitment to a collective endeavor to focus on customers and continuously improve the business and care processes within the organization’.*

1. The treatment plan is discussed with each patient and a joint agreement is made with patients about the goals that will be pursued in their care process.
2. Results of patient satisfaction surveys are shared on a structural basis with all employees.
3. Every employee continuously strives to satisfy patients.
4. Hospital staff on all levels communicates in terms of processes, teams and process performance.
5. The continuous improvement of care processes is an important principle in our hospital.
6. Health care professionals and managers work together to deliver the right care, on the right time and at the right place to patients.
7. Each employee has a role in the organization of care processes and together we organize the entire care process for patients.
